# Trends and patterns in pulmonary arterial hypertension-associated hospital admissions among methamphetamine users: a decade-long study

**DOI:** 10.3389/fcvm.2024.1445193

**Published:** 2024-10-28

**Authors:** Amanda Husein, Jolie Boullion, Md Ismail Hossain, Diensn Xing, Md Tareq Ferdous Khan, Md. Shenuarin Bhuiyan, Gopi K. Kolluru, Md Mostafizur Rahman Bhuiyan, Nicholas E. Goeders, Steven A. Conrad, John A. Vanchiere, A. Wayne Orr, Christopher G. Kevil, Mohammad Alfrad Nobel Bhuiyan

**Affiliations:** ^1^ Department of Medicine, Louisiana State University Health Sciences Center at Shreveport, Shreveport, LA, United States; ^2^ Department of Mathematics and Statistics, Cleveland State University, Cleveland, OH, United States; ^3^ Department of Molecular and Cellular Physiology, Louisiana State University Health Sciences Center at Shreveport, Shreveport, LA, United States; ^4^ Department of Pathology and Translational Pathobiology, Louisiana State University Health Sciences Center at Shreveport, Shreveport, LA, United States; ^5^ Department of Pediatric Cardiology, Bangabandhu Sheikh Mujib Medical University, Dhaka, Bangladesh; ^6^ Department of Pharmacology, Toxicology & Neuroscience, Louisiana State University Health Sciences Center at Shreveport, Shreveport, LA, United States; ^7^ Department of Psychiatry and Behavioral Medicine, Louisiana State University Health Sciences Center at Shreveport, Shreveport, LA, United States; ^8^ Louisiana Addiction Research Center, Louisiana State University Health Sciences Center at Shreveport, Shreveport, LA, United States; ^9^ Department of Pediatrics, LSU Health Sciences Center Shreveport, Shreveport, LA, United States

**Keywords:** pulmonary arterial hypertension, methamphetamine, substance use disorder, methamphetamine use disorder, disparity

## Abstract

**Background:**

Pulmonary arterial hypertension (PAH) is a rare, chronic, progressive form of pulmonary hypertension in which increased arterial pressure causes remodeling of the arterial system and is associated with heart failure. Methamphetamine is a stimulant that has recently become a focus in PAH research, but the recent trends and demographics of this cohort of patients are not known. The study aimed to analyze the overall trends and demographics of PAH patients with and without concurrent methamphetamine usage.

**Methods:**

The study used the National Inpatient Sample (NIS), Healthcare Cost and Utilization Project (HCUP), and Agency for Healthcare Research and Quality (AHRQ) from 2008 to 2020 to calculate nationally weighted estimates for both conditions by ICD-9 and ICD-10 diagnosis codes. We used several statistical measures, including descriptive statistics with design-based chi-square and *t*-tests, trend analysis with Cochran-Armitage test, generalized linear models, and other data preprocessing measures.

**Results:**

A significant increase was evident in patients with pulmonary arterial hypertension (PAH) and concurrent methamphetamine use (9.2-fold). Most of the hospitalized patients were males (59.16%), aged 41–64 (45.77%), White (68.64%), from the West (53.09%), with Medicaid (50.48%), and with median income <$25,000. The rate of increase over the period was higher for males (11.8-fold), race (not sure which race; please check and modify), aged 41–64 (11.3-fold), and in the South (15.1-fold). An overall adjusted prevalence ratio (PR) for PAH hospitalizations among concurrent methamphetamine users was 32.19 (CI = 31.19–33.22) compared to non-users. With respective reference categories, the significantly higher PR was evident for males, patients aged 41–64, White, with Medicare, median income <$25,000, all regions compared to Northeast, length of hospital stays, and conditions, including chronic pulmonary disease, diabetes, hypertension, obesity, and peripheral vascular disorders.

**Conclusion:**

This study reveals a national overall and demographic-specific trend of increasing PAH with concurrent methamphetamine usage and associated factors. The findings may help to understand the current patterns and identify the vulnerable sociodemographic cohorts for further research and to take appropriate policy measures.

## 1 Introduction

Pulmonary hypertension is defined as an increase in the mean arterial pressure (MAP) of the lungs by greater than 20 mmHg (1, 2). Cardiopulmonary pathologies that cause an increase of pressure in the lungs are categorized into 5 groups: pulmonary arterial hypertension, pulmonary hypertension due to center heart disease, pulmonary hypertension due to lung disease, pulmonary hypertension due to blood clots in the lungs, and pulmonary hypertension due to unknown causes (3). Pulmonary arterial hypertension (PAH) is one of the most damaging forms of pulmonary hypertension, with an estimated 10.6 per 1 million adults in the US affected (4). PAH is defined as increased MAP in the pulmonary arteries, measured by right heart catheterization, in the absence of cardiac disease, abnormal lung pathology, and thromboembolic causes (5). Increased arterial pressure in PAH causes remodeling of the arterial system, increasing vascular resistance and right ventricular afterload, which may eventually develop into heart failure (6).

PAH is characterized as arterial remodeling in pulmonary vasculature that leads to vasculature resistance, right heart failure, and potential death. The vasculature displays medial hypertrophy and both intimal and adventitial proliferation. These changes may lead to concentric sclerosis from the endothelial cells and pulmonary arterial smooth muscle cells migrating and proliferating, causing endothelial dysfunction and vasoconstriction (7). Endothelial cells can create growth factors that stimulate matrix deposition and smooth muscle hypertrophy, leading to vascular formations called plexiform lesions, the hallmark sign of PAH remodeling (7). Pharmacologic treatments of PAH thus target these specific pathways to resist the vasoconstrictive causes and promote vasodilation (8). The most recent pharmacotherapies focus on targeting pathways such as TGF-B and tyrosine kinases to primarily inhibit the inflammatory cell proliferation in the vasculature (9, 10).

The pathology of drug-induced PAH is similar to idiopathic PAH, with both having plexiform lesions as signs of remodeling (11). Many drug compounds have been associated with PAH, including anorexigens, SSRIs, oral contraceptives, and stimulants. Methamphetamine is a stimulant that has become a focus in PAH research and is now labeled in the “definite” category as a causative agent of PAH. Although the pathology is similar to idiopathic PAH, methamphetamine-induced PAH has been shown to cause severe consequences in long-term damage and functional status (12). Methamphetamine has been shown to have harmful overall effects, such as CNS toxicity, cardiomyopathy, cellular remodeling, and its general addictive quality due to interfering with the long-term production of the brain's dopamine (13–15). Among patients with cardiovascular disease (CVD), patients with prior methamphetamine usage have been shown to develop the disease eight years earlier than patients without prior methamphetamine usage (16). PAH among meth users have also been shown to have significantly higher right atrial pressure and lower stroke volume (17). The national level of methamphetamine-associated cardiac failure has risen since 2002 (18). On the cellular level, a prior study using rat models revealed that methamphetamine can also affect the function of the mitochondria and stimulate autophagy (15). Methamphetamine is now also classified as a substance with numerous physical and psychotic side effects (19) and its usage has increased in mentally vulnerable populations with higher rates of suicidal ideation (20).

A previous study found that 28.9% of patients with “idiopathic PAH” had a history of stimulant use, compared to 3.8% of patients who had PAH due to well-known risk factors, such as genetics, hypertension, arterial disease, or connective tissue disease (11). Pathologic lung samples taken from PAH among meth users showed vascular changes similar to those in other PAH patients, including the plexiform lesions and slit-like vascular channel with occlusive disease. Serotonin has been suspected to be a cause of PAH among meth users due to its ability to stimulate pulmonary smooth muscle cells, causing arterial narrowing and increased pressures (21). With the concerning increases in methamphetamine-related overdose deaths and methamphetamine-related hospitalizations that have led the US to declare methamphetamine an emerging drug threat, it is important to re-examine the trends in PAH with and without methamphetamine use (22–25). Thus, the focus of this study is to analyze the overall trends and demographics of PAH alone in comparison to PAH with concurrent methamphetamine usage.

## 2 Methods

To analyze the yearly trends of PAH and methamphetamine use, this study utilized the National Inpatient Sample, Healthcare Cost and Utilization Project, and Agency for Healthcare Research and Quality (AHRQ) collected between 2008 and 2020. The NIS is the US's largest all-payer inpatient care database, representing about 7 million hospital stays annually. Recent modifications to the NIS improved the sample's representativeness and the accuracy and stability of weighted national estimates. This new approach was also applied to existing data, allowing trend analysis across long periods. The new NIS data is formatted so that every datum corresponds to a unique hospitalization and includes details on more than 100 clinical characteristics, including patient demographics and primary and secondary (up to 24) diagnoses. Using the ICD-9 and ICD-10 codes listed in Supplementary Tables S3, S4 in the supplemental information document, we identified all adults (>18 years of age) hospitalized with a primary or secondary diagnosis of PAH and methamphetamine use and their associated sociodemographic characteristics. All tests have been performed at 5% level of significance and reported Cis are with 95% confidence level. The NIS database was deidentified; hence, our study was exempt from requiring an institutional review board (IRB) approval.

### 2.1 Statistical analysis

For the statistical analysis, we followed the guidelines for the AHRQ. All statistical tests were conducted using the software R (version 4.2.1). Survey-specific statements with hospital and patient-level weights were utilized for national estimates, and trend weight was used to produce national estimates for trend analysis. The NIS year's hospital discharges all used equal discharge weights each year. Thus, the trend weight files were combined with the original NIS files by year and hospital ID. The frequencies and percentages of categorical variables were summarized for unweighted (raw hospital admission data) and weighted (nationally representative data) hospital admissions. We used design-based chi-squared tests for categorical variables, and design-based *t*-tests for continuous variables to account for NIS sample design and sample weight (26). To observe any patterns in the missing data, we employed Little's MCAR (missing completely at random). A nonsignificant *p*-value (*p* > 0.05) was used to detect random missing, and the missing value was less than 5%. Thus, no imputation was performed. We utilized propensity score matching with the nearest neighbor method to match the case and control for methamphetamine users (27). The prevalence ratio and 95% confidence interval were calculated using a generalized linear model with a binomial link function (26). The model was adjusted for gender, age, race, primary payer, median household income, length of hospital stays, region, anemia, arthritis, chronic pulmonary disease, congestive heart failure, coagulopathy, depression, diabetes, liver disease, hypertension, obesity, peripheral vascular disorders, pulmonary circulation disorders, and renal failure. Multicollinearity among the variables was investigated using the variance inflation factor (VIF). We used eta-squared to measure the proportion of the total variability in a dependent variable that is explained by the variability in an independent variable. Eta-squared is calculated by dividing the sum of squares associated with an effect (e.g., treatment) by the total sum of squares (28). The *p*-value was determined by linear trend analysis, and the trend, stratified by age, sex, race, and region, was compared using the Cochran-Armitage trend test. There are existing descriptions of the design and analytical guidelines for NIS data (29, 30). The Healthcare Cost and Utilization Project criteria were followed for performing the analysis (30).

## 3 Results

Between 2008 and 2020, there were 114,177,810 reported hospitalizations for PAH in the US without concurrent usage of methamphetamine and 1,104,142 hospitalizations for PAH with concurrent methamphetamine usage.

### 3.1 Demographic characteristics among idiopathic PAH vs. PAH among meth users hospitalizations

Table 1 summarizes the PAH hospitalizations with and without concurrent methamphetamine use in the US between 2008 and 2020. Significantly (*p* < 0.05) more hospitalizations due to idiopathic PAH alone were seen among female patients (51.97%, CI = 51.96–51.98) than among male patients (48.03%, CI = 48.02–48.04). For hospitalizations due to PAH with concurrent methamphetamine, 59.16% (CI = 59.07–59.25) were male, and 40.84% (CI = 40.75–40.93) were female.

**Table 1:** Sociodemographic characteristics (weighted) of hospitalized patients with PAH and status of concurrent methamphetamine use from 2008 to 2020 in the US^a^.

Characteristics	Group	PAH with methamphetamine use		PAH without methamphetamine use		*p*-value
		Weighted *n* (%)	(95% confidence interval)	Weighted *n* (%)	(95% confidence interval)	
		*N* = 1,104,142		*N* = 114,177,810		
Sex	Male	653,076 (59.16)	(59.07–59.25)	54,835,651 (48.03)	(48.02–48.04)	<0.05
	Female	450,802 (40.84)	(40.75–40.93)	59,328,378 (51.97)	(51.96–51.98)	
Age	18–25 years	117,780 (10.67)	(10.61–10.72)	1,321,806 (1.16)	(1.16–1.16)	<0.05
	26–40 years	414,303 (37.43)	(37.43–37.61)	5,559,560 (4.87)	(4.87–4.87)	
	41–64 years	505,367 (45.77)	(45.68–45.86)	38,109,382 (33.38)	(33.37–33.39)	
	≥65 years	66,689 (6.04)	(6.00–6.08)	69,187,059 (60.60)	(60.59–60.60)	
Race/ethnicity	White	7,19,223 (68.64)	(68.56–68.73)	80,476,632 (75.60)	(75.59–75.61)	<0.05
	Black	100,189 (9.56)	(9.51–9.62)	15,043,765 (14.13)	(14.13–14.14)	
	Hispanic (ethnicity)	152,850 (14.59)	(14.52–14.66)	6,567,027 (6.17)	(6.16–6.17)	
	Asian or Pacific Islander	28,177 (2.69)	(2.66–2.72)	1,542,218 (1.45)	(1.45–1.45)	
	Native American	19,859 (1.90)	(1.87–1.92)	577,557 (0.54)	(0.54–0.54)	
	Other	27,452 (2.62)	(2.59–2.65)	2,241,879 (2.11)	(2.10–2.11)	
US Region	Northeast	41,877 (3.79)	(3.76–3.83)	21,261,991 (18.62)	(18.61–18.63)	<0.05
	Midwest	185,691 (16.82)	(16.75–16.89)	28,820,342 (25.24)	(25.23–25.25)	
	South	290,336 (26.30)	(26.21–26.38)	44,646,423 (39.10)	(39.09–39.11)	
	West	586,237 (53.09)	(53.0–53.19)	19,449,051 (17.03)	(17.03–17.04)	
Primary payer	Medicare	202,831 (18.42)	(18.35–18.49)	75,691,243 (66.40)	(66.39–66.40)	<0.05
	Medicaid	555,906 (50.48)	(50.39–50.57)	11,760,803 (10.31)	(10.31–10.32)	
	Private Insurance	133,985 (12.17)	(12.11–12.23)	20,622,134 (18.09)	(18.08–18.10)	
	Self-pay	148,388 (13.47)	(13.41–13.54)	2,942,186 (2.58)	(2.58–2.58)	
	No charge	8,259 (0.75)	(0.73–0.77)	291,847 (0.26)	(0.26–0.26)	
	Other	51,891 (4.71)	(4.67–4.75)	2,692,341 (2.36)	(2.36–2.36)	
Median household income ($)	<25,000	376,473 (37.47)	(37.38–37.57)	34,526,400 (30.84)	(30.83–30.85)	<0.05
	26,000–50,000	289,714 (28.84)	(28.75–28.93)	30,279,683 (27.05)	(27.04–27.06)	
	51,000–75,000	217,979 (21.70)	(21.62–21.78)	26,260,495 (23.46)	(23.45–23.47)	
	76,000–100,000	120,440 (11.99)	(11.93–12.05)	20,875,638 (18.65)	(18.64–18.66)	

^a^ The designed-based chi-square for categorical variables and *t*-tests for continuous variables were applied to obtain the *p*-values.

Hospitalizations due to PAH without concurrent methamphetamine use were most prevalent among patients older than age 64 (60.60%, CI = 60.69–60.60), followed by ages 41–64 (33.38%, CI = 33.37–33.39), ages 26–40 (4.87%, CI = 4.87–4.87), and ages 18–24 (1.16%, CI = 1.16–1.16). In contrast, hospitalizations due to PAH among meth users were increased among middle-aged patients. The majority occurred at ages 41–64 (45.77%, CI = 45.68–45.86), followed by ages 26–40 (37.52%, CI = 37.43–37.61), ages 18–25 (10.67%, CI = 10.61–10.72), and older than 64 years of age (6.04%, CI = 6.00–6.08).

By race/ethnic group, non-Hispanic White (henceforth White) patients comprised the majority of hospitalizations (75.60%, CI = 75.59–75.61) for PAH alone, followed by non-Hispanic Black (henceforth Black) patients (14.13%, CI = 14.13–14.14). For hospitalizations of PAH with concurrent methamphetamine usage, although White patients retained its majority (68.64%, CI = 68.56–68.73), Hispanic patients were in the next majority group (14.59%, CI = 14.52–14.66), followed by Black patients (9.56%, CI = 9.51–9.62). A similar race was noted between both groups, except PAH hospitalizations among meth users were more commonly attributed to Hispanic patients than Black patients.

Regarding primary payer, the highest amount of hospitalizations (66.40%, CI = 66.39–66.40) due to PAH alone were Medicare patients, followed by private insurance patients (18.09%, CI = 18.08–18.10), Medicaid patients (10.32%, CI = 10.31–10.320, self-pay patients (2.58%, CI = 2.58–2.58), patients with no charge (0.26%, CI = 0.26–0.26), and those who did not report (2.36%, CI = 2.36–2.36). The highest number of hospitalizations due to PAH with concurrent methamphetamine were Medicaid patients (50.48%, CI = 50.39–50.57), followed by Medicare patients (18.42%, CI = 18.35–18.49), self-pay patients (13.47%, CI = 13.41–13.54), private insurance patients (12.17%, CI = 12.11–12.23), patients with no charge (0.75%, CI = 0.73–0.77), and patients who reported “Other” (4.71%, CI = 4.67–4.75).

The hospitalized patients’ distribution by primary insurance showed most patients with PAH and without concurrent methamphetamine use had Medicare (66.4%, CI = 66.39–66.40) as their primary insurance compared to Medicaid for PAH with concurrent methamphetamine use (50.48%, CI = 50.39–50.57). Private insurance (18.09%, CI = 18.08–18.10), followed by Medicaid (10.32%, CI = 10.31–10.32), was the second primary insurance source for Patients with PAH alone; for patients with PAH and concurrent methamphetamine, Medicare (18.42%, CI = 18.35–18.49) followed by self-pay patients (13.47%, CI = 13.41–13.54) were the next primary sources.

Regarding US geographic region, the highest number of hospitalizations due to PAH alone were located in the South United States (39.10%, CI = 39.09–39.11), followed by the Midwest region (25.24%, CI = 25.23–25.25. For hospitalizations of PAH with concurrent methamphetamine usage, patients in the West made up the majority (53.09%, CI = 53.00–53.19), followed by the South (26.30%, CI = 26.21–26.38).

Regarding household income, the number of hospitalizations due to PAH alone was inversely correlated to household income. Among patients with PAH alone, the most significant number of hospitalizations occurred in patients with an income of <$25,000 (30.84%, CI = 30.83–30.85), followed by <$50,000 (27.05%, CI = 27.04–27.06). The highest number of hospitalizations due to PAH with concurrent methamphetamine also followed an inverse correlation, 37.47% (CI = 37.38–37.57) for <$25,000 income, followed by 28.84% (CI = 28.75–28.93) for <$50,000 income.

### 3.2 Temporal trend of pulmonary arterial hypertension hospitalizations without methamphetamine use

**Figure 1:**
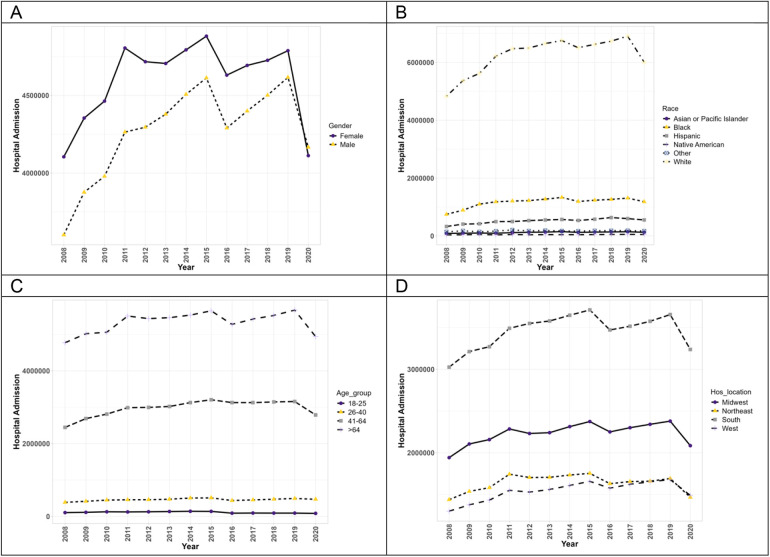
The hospitalization trends of PAH patients without concurrent methamphetamine usage by sex **(A)**, race **(B)**, age **(C)**, and geographic region **(D)** from 2008 to 2020 in the US.

Figure 1 exhibits the trends of hospitalizations of patients with PAH without concurrent methamphetamine use. From 2008 to 2020, hospitalizations associated with PAH without concomitant methamphetamine use increased from 7,706,820 to 8,278,579 (1.1-fold, *p* = 0.07). Throughout this period, PAH-related hospitalizations without concomitant methamphetamine use of both male and female patients initially increased from 2008 to 2015 and later fluctuated and down-trended from 2015 to 2020. Female patients showed a higher prevalence every year than male patients; however, male patients had an overall statistically significant (*p* < 0.05) upward trend (1.16-fold increase) compared to the female patient fluctuation (*p* = 0.45, 1-fold increase). Hospitalizations by race significantly increased for all races (*p* < 0.05). White patients had a substantially higher yearly prevalence compared to other races. However, the rate of change was not the highest (1.2-fold), compared to the overall rate of change in Hispanic patients (1.7-fold increase), Black patients (1.6-fold increase), Asian/Pacific Islander (1.4-fold increase), and Native Americans (1.2-fold increase). Patients aged 26–40 and 41–64 had a statistically significant upward trend throughout the years (*p* < 0.05) with overall increases of 1.2-fold and 1.1-fold, respectively). Patients aged 18–25 and greater than 64 years had insignificant trends (*p* = 0.05 and *p* = 0.15, respectively) with an overall decrease (0.8-fold increase). Patients located in the West showed the only significant (*p* < 0.05) upward trend of PAH (1.2-fold increase), while the other regions (South, Midwest, and Northeast) showed insignificant trend (*p* > 0.05) with some fluctuations throughout the years (1.1-fold increase, 1.1-fold increase, and 1.1-fold increase, respectively).

### 3.3 Temporal trend of pulmonary arterial hypertension hospitalizations with concurrent methamphetamine use

**Figure 2:**
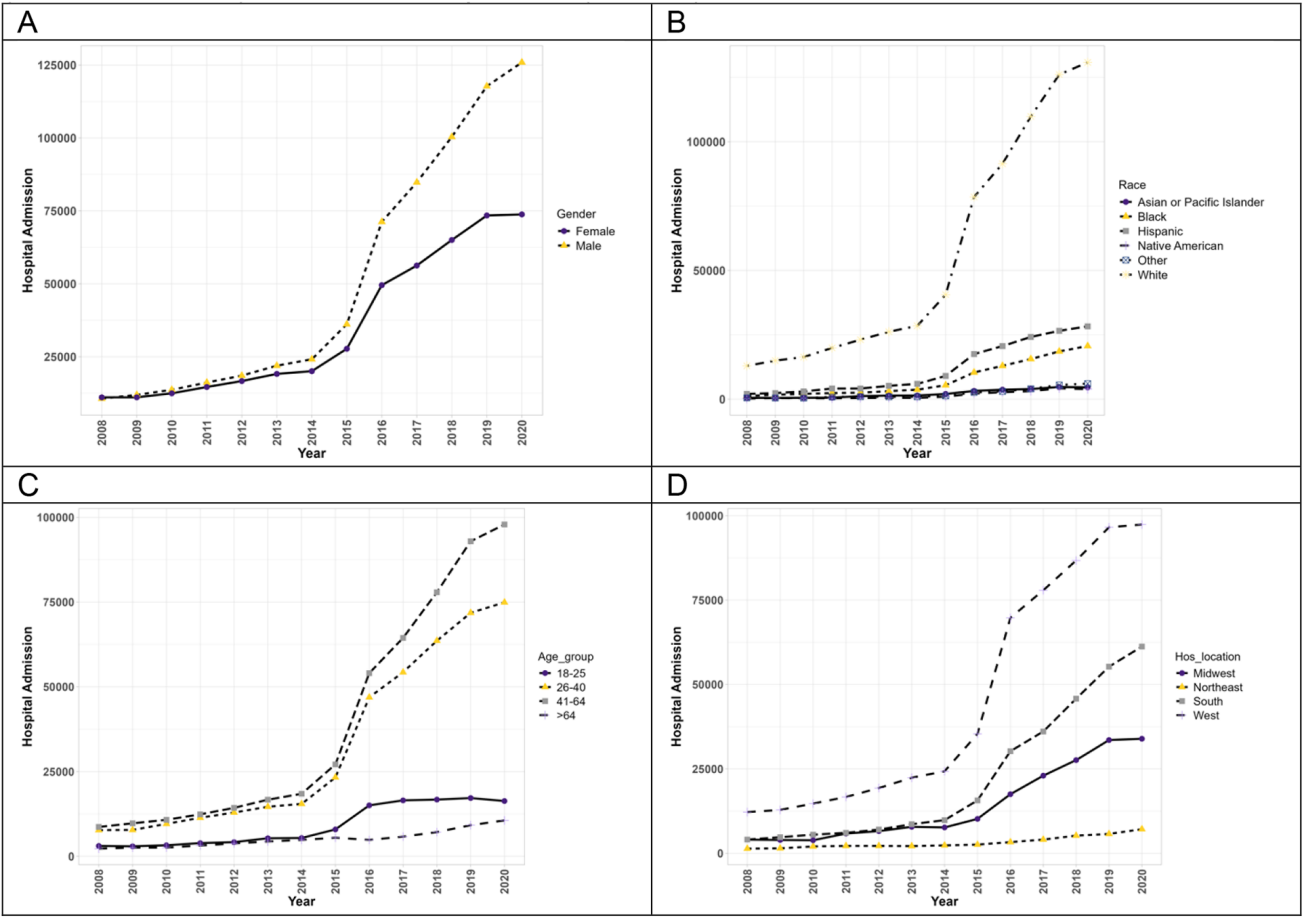
The hospitalization trends of PAH patients with concurrent methamphetamine usage by sex **(A)**, race **(B)**, age **(C)**, and geographic region **(D)** from 2008 to 2020 in the US.

Figure 2 exhibits the trends of hospitalizations of patients with PAH with concurrent methamphetamine use. From 2008 to 2020, hospitalizations of PAH among meth users increased (9.2-fold) from 21,738 in 2008 to 199,715 in 2020. Throughout this period, hospitalizations of both male and female patients increased significantly (*p* < 0.05). Moreover, male patients of PAH among meth users showed a higher prevalence each year and a higher overall increase throughout the years (11.8-fold increase vs. 6.7-fold increase). Hospitalizations statistically increased for all races (*p* < 0.05). Native American patients had the highest overall increase throughout the years (20.4-fold increase), followed by Black patients (16.4-fold), Hispanic patients (13.9-fold), White patients (10.1-fold), and Asian/Pacific Islander patients (8.5-fold). The trend in race for PAH patients with concurrent methamphetamine use differed slightly from the trend for PAH patients without concurrent methamphetamine use, where Hispanic patients’ overall trend was higher than Black patients’ overall trend. With respect to hospitalizations by age, the trend differed slightly from PAH patients without methamphetamine use, with all patients showing a statistically significant upward trend throughout the years (*p* < 0.05). Patients aged 41–64 had the highest overall increase (11.3-fold), followed by patients aged 26–40 (9.7-fold), aged 18–25 (5.3-fold), and aged more significant than 64 years (4.7-fold). In terms of geographic regions, PAH patients with concurrent methamphetamine use in all regions of the US showed a statistically significant upward trend (*p* < 0.05), with patients in the South having the highest overall increase throughout the years (15.1-fold increase), followed by Midwest patients (8.4-fold), West patients (8.0-fold increase), and Northeast patients (5.4-fold).

Overall, hospitalizations of PAH among meth users from 2008 to 2020 demonstrated a 1.53 adjusted prevalence ratio (PR) in methamphetamine users compared to non-methamphetamine users (Table 2). When examining mental health disorder-related hospitalizations from 2008 to 2020 with concurrent methamphetamine use, female patients showed a higher adjusted PR (1.53, CI = 1.51–1.56) compared to males. Patients aged 18–25 showed the highest adjusted PR, with those aged 26–40 and 41–64 showing a comparable but lower adjusted PR of 0.89 (CI = 0.87–0.92) and 0.71 (CI = 0.69–0.73) compared to patients aged 18–25. White patients showed the highest adjusted PR, with Native American patients showing the next highest adjusted PR (0.73, CI = 0.69–0.78) compared to White patients. When examining household income, those with income of $50,000–64,999, $65,000–85,999, or >$86,000 showed a similar adjusted PR of 1.02 (CI = 1–1.04), 1.06 (CI = 1.03–1.08) and 1.09 (CI = 1.06–1.12), respectively compared to those with household income <$50,000. When examining regions, the Midwest showed the highest adjusted PR (1.24, CI = 1.18–1.30) compared to the Northeast, with the South showing a similar adjusted PR (0.90, CI = 0.86–0.95) compared to the Northeast.

**Table 2:** Adjusted PR of PAH hospitalizations with concurrent methamphetamine use with 95% CI.

Characteristics	Group	PAH hospitalizations with concurrent methamphetamine use	
		Adjusted prevalence ratio	95% confidence interval
Methamphetamine user	Yes	32.19	(31.19, 33.22)
Sex	Male	1 [Reference]	
	Female	0.77	(0.72, 0.79)
Age	18–25 years	0.76	(0.72–0.80)
	26–40 years	0.90	(0.86, 0.95)
	41–64 years	1.33	(1.27, 1.40)
	≥65 years	1 [Reference]	
Race/ethnicity	White	1 [Reference]	
	Black	0.82	(0.79, 0.84)
	Hispanic (ethnicity)	0.75	(0.73, 77)
	Asian or Pacific Islander	0.73	(0.68, 0.77)
	Native American	0.91	(0.85, 0.97)
	Other	0.81	(0.77, 0.86)
Primary payer	Medicare	1 [Reference]	
	Medicaid	0.92	(0.90, 0.95)
	Private insurance	0.63	(0.61, 0.65)
	Self-pay	0.87	(0.84, 0.90)
	No charge	0.74	(0.67, 0.83)
	Other	0.86	(0.82, 0.91)
Median household income($)	<25,000	1 [Reference]	
	25,000–50,000	0.95	(0.93, 0.97)
	51,000–75,000	0.90	(0.88, 0.92)
	76,000–100,000	0.80	(0.78, 0.83)
US Region	Northeast	1 [Reference]	
	Midwest	1.51	(1.42, 1.60)
	South	1.48	(1.40, 1.57)
	West	1.91	(1.81, 2.02)
Length of hospital stay	0–3 days	1 [Reference]	
	4–6 days	1.32	(1.29, 1.34)
	7–9 days	1.41	(1.37, 1.46)
	10–12 days	1.34	(1.28, 1.40)
	>12 days	1.26	(1.21, 1.31)
Chronic pulmonary disease	Yes	6.00	(5.84, 6.19)
Diabetes	Yes	1.17	(1.14, 1.20)
Hypertension	Yes	1.23	(1.21, 1.26)
Obesity	Yes	1.80	(1.75, 1.85)
Peripheral vascular disorders	Yes	1.61	(1.51, 1.70)

An overall adjusted prevalence ratio (PR) for PAH hospitalizations among concurrent methamphetamine users was 32.19 (CI = 31.19–33.22) compared to non-users (Table 2). Female patients showed a lower adjusted PR (0.77, CI = 0.72–0.79) than males. Patients aged 41–64 showed the highest adjusted PR (1.33, CI = 1.27–1.40) compared to the reference cohort 65 years or older, and those aged 26–40 and 41–64 showed lower adjusted PRs of 0.90 (CI = 0.86–0.95) and 0.71 (CI = 0.69–0.73), respectively. Compared to White patients, other races showed lower PRs ranging from 0.73 to 0.91. With the increase in patients’ income, the PR decreases. Compared to patients’ median income of less than $25,000, the PR for the highest median income of $76,000–100,000 was 0.80 (CI = 0.78–0.83). By regions, the West showed the highest adjusted PR of 1.91 (CI = 1.81–2.02), followed by the Midwest of 1.51 (CI = 1.42–1.60) and the South of 1.48 (CI = 1.40–1.57) compared to the Northeast. Patients with other insurances showed significantly lower PRs, ranging from 0.63 to 0.92, compared to Medicare as the primary source of insurance. With the increase in hospital stays, the PRs were also increased significantly. Chronic pulmonary disease is associated with increasing prevalence 6 times (CI = 5.84–6.19). Similarly, the history of diabetes (PR = 1.17, CI = 1.14–1.20), hypertension (PR = 1.23, CI = 1.21–1.26), obesity (PR = 1.80, CI = 1.75–1.85) and peripheral vascular disorders (PR = 1.61, CI = 1.51–1.70) were also associated with increasing prevalence.

## 4 Discussion

According to NIS data from 2008 to 2020, a total of 0.9% of PAH hospitalizations were concurrent methamphetamine users. During this period, there has been a significant overall increase in hospitalizations of PAH among meth users (9.2-fold). For PAH hospitalizations alone, the overall increase was only 1.1-fold and not statistically significant, likely due to the large decline in hospitalizations for PAH alone in 2015 and 2019. The disparity seen between the increases in PAH-related hospitalizations with concurrent methamphetamine use compared to those without concurrent methamphetamine use are paralleled by the increasing widespread use of methamphetamine in the US (31) and overall increase in drug use-related hospital admissions in the nation (32), suggesting that, in this patient population, the increase in methamphetamine use is contributory to the increase in PAH-related hospitalizations. The demographic groups that had the highest overall prevalence in PAH hospitalizations without concurrent methamphetamine use from 2008 to 2020 were patients who are White, female, those older than age 64, those in the Southern US, Medicare patients, and those with a salary of less than $25,000.

The results showed a significant disparity in the prevalence of hospitalizations within each demographic. These disparities are consistent in a previous methamphetamine study that solely focused on the differing demographics of methamphetamine users in the US in the same ten-year time frame (33). Previous studies analyzing the demographics of idiopathic PAH patients show the predominant sex was consistently female and the mean age was 53, but have not analyzed race, geographic region, or financial/insurance statuses (34, 35). The demographic groups that have the highest overall trend but not the highest overall prevalence reveal the change in the epidemiology of methamphetamine use and associated PAH.

Males had a higher overall prevalence of PAH with concurrent methamphetamine use compared to females and a higher overall increase in trend. Previous studies have shown that methamphetamine use occurs more commonly among males than females (36, 37). Studies have also shown that idiopathic PAH has a higher incidence in females due to the effects of estrogen on the cardiovascular system (38). These results of previous studies help to suggest that for this study, methamphetamine use itself is suspected to be responsible for the higher PAH in males. Given the effects of methamphetamine shown to decrease systolic function, the combination of the cardiotoxic effects of PAH and methamphetamine can lead to a significant risk for cardiac failure. With this in mind, it is important for physicians to mainly evaluate male methamphetamine user patients for cardiac function and screen for any risk factors, such as hypertension, diabetes, or hyperlipidemia, that may lead to higher chances of cardiac failure.

Both hospitalizations due to PAH alone and PAH among meth users showed significant differences among age groups. While hospitalizations due to PAH without methamphetamine use were most common in those ages ≥65 years and 41–64 years, hospitalizations due to PAH with methamphetamine use were most common in those 41–64 and 26–40 and least common with those ages ≥65 years. This suggests that when PAH is combined with comorbid methamphetamine use, disease severity is likely greater, as younger patients are being affected. The low prevalence in those aged ≥65 could also suggest that the disease is so severe that survival with both conditions is lower. However, this could be due to decreased prevalence of methamphetamine use in that population. Furthermore, the age group with the highest increase in trend was 41–64, followed by the younger age groups of 26–40 and 18–25, respectively. These increases further our point that comorbid PAH and methamphetamine use are a growing issue and support our idea that these patients are affected at younger ages. This is also likely related to age differences seen in methamphetamine use, as studies have shown that those ages 26–64 make up the majority of methamphetamine users (31, 37). Physicians are more likely to assess for PAH in older patients due to the disease normally occurring with chronic conditions of CAD or dyspneic effects (39). Since methamphetamine use prevalence is highest in the middle-aged population, younger patients with methamphetamine use should be counseled on their higher PAH risk compared to the general population.

White patients had the highest prevalence of PAH with concurrent methamphetamine use. For PAH alone, there are different pathogenic causes for PAH development among each race. White patients are more likely to develop PAH from genetics or drug/toxin use. Hispanic patients are more likely to develop PAH from congenital heart disease, and Black patients from connective tissue disease (40). Thus, White patients are expected to have the highest prevalence of drug-induced PAH in the US. However, regarding overall trend rather than prevalence, Native American patients had the highest overall increase from 2008 to 2020 in the US. These results highlight the epidemiological shift in methamphetamine usage. Although historically, methamphetamine has been primarily used by White persons, the drug is becoming increasingly more popular in other racial groups, such as those who are Native American. Previous studies have shown that methamphetamine use is higher among those who are Native American than any other racial group in the United States, with an earlier onset age as well, which may explain the reason for Native American patients having the highest increase in PAH with concurrent methamphetamine use (41). Cardiovascular disease is one of the highest causes of mortality among the Native American population (42). With this already high risk, it is important to be aware of the potential cardiovascular danger in Native American methamphetamine users.

The South had an overall higher prevalence of PAH without concurrent methamphetamine usage, while the West had the highest overall prevalence of PAH with concurrent methamphetamine usage. This aligns with previous studies that have shown overall higher PAH among meth users in the general southwestern region of the US (43). However, in terms of the trends in PAH hospitalizations with concurrent methamphetamine use from 2008 to 2020 in the US, the South had the highest overall trend increase, which may correlate with the rise of illicit drug use in formerly low prevalence areas (44). The South United States has been shown to have a higher prevalence of a multitude of chronic diseases that increase morbidity, including obesity, diabetes, hypertension, and cardiovascular disease. With methamphetamine's damaging cardiovascular effects, the increase in the overall methamphetamine trend in the South can worsen a population already vulnerable to cardiovascular disease (45).

Physicians can incorporate this knowledge as they counsel patients about drug use, including patients with no current risk factors who may simply have demographic or social risk factors. Physicians should be educated both on the signs and symptoms of methamphetamine use and PAH to know when a drug screening may be necessary. Acute symptoms of methamphetamine include increased energy, euphoria, decreased need for sleep, excessive talking, weight loss, sweating, tightened jaw muscles, grinding teeth, and loss of appetite. Methamphetamine may also cause psychotic symptoms (such as hallucinations, unusual behavior, and suspiciousness), psychomotor symptoms (such as tension, excitability, and motor hyperactivity), and affective symptoms (such as depression or suicidality) (46). On a larger scale, the results of this study can be utilized to understand better the target audience that would most benefit from a drug intervention or education program. Outside of the one-on-one patient interaction, this study reveals the need for an overall national public health movement aimed toward stopping the continuous rise in both PAH and methamphetamine use (44). This public health movement can aim towards educating physicians as well as psychiatric and substance use treatment centers and continuing to track and release records of drug use data. A previous study suggests options for an effective public health solution is data documentation in programs such as the CDC's Overdose Data to Action (OD2A), the State Unintentional Drug Overdose Reporting System (SUDORS), the Drug Overdose Surveillance and Epidemiology (DOSE), or National Drug Early Warning System (NDEWS) (25). These programs support state, territorial, county, or city health department's use of methamphetamine overdose data collection for prevention and response efforts. Overall, our results further support the movement for the documentation, research, and education on PAH among meth users to decrease the national disease trend.

The findings of this study have several important implications for public health policies and interventions. First, the significant increase in PAH hospitalizations among specific sociodemographic groups—particularly Hispanics, males, individuals aged 41–64, and those living in the South—highlights the need for targeted public health campaigns aimed at these vulnerable populations. Proper education and outreach programs can raise awareness about the risks of methamphetamine use and its link to PAH, encouraging preventive behaviors and early intervention. Additionally, these trends suggest the need for more comprehensive healthcare policies that address both substance use and disease management, particularly in regions and demographics most affected. Expanding access to addiction treatment services and ensuring adequate healthcare resources in high-risk communities could help mitigate the growing burden of PAH among methamphetamine users. Moreover, these findings should prompt policymakers to consider revising current strategies and policies related to drug use, healthcare access, and disease prevention. By integrating these insights into national and local health initiatives, public health authorities can better address the dual challenges of substance abuse and its long-term health consequences, ultimately reducing the incidence of PAH and improving health outcomes in at-risk populations.

## 5 Strength and limitations

One of the primary advantages is its large sample size, which enhances the statistical power of studies and allows for more robust and generalizable findings across diverse populations. Additionally, the NIS provides comprehensive coverage of hospitalizations across the United States, capturing data from a wide range of healthcare facilities. This broad representation enables the investigation of national trends and patterns in hospital admissions. Furthermore, the NIS allows for the analysis of a wide variety of health conditions and procedures, facilitating large-scale studies on healthcare utilization, outcomes, and disparities. However, the study also had limitations. One key limitation is that the NIS data primarily focuses on hospitalizations, making it challenging to capture outpatient interactions or post-discharge outcomes, potentially leading to an underrepresentation of overall healthcare utilization and outcomes. Additionally, the inability to identify patients with multiple admissions introduces the possibility of counting the same patient more than once, which could skew the results. Other limitations include potential biases and confounding factors inherent in observational data. For instance, the NIS lacks detailed clinical data, such as specific biological or behavioral factors, that could influence both methamphetamines use and the development of pulmonary arterial hypertension. This introduces the possibility of confounding variables that are not accounted for in the analysis. Future research is necessary to address these gaps by incorporating more detailed clinical data and tracking patients across various care settings.

## Data Availability

Publicly available datasets were analyzed in this study. This data can be found here: https://hcup-us.ahrq.gov/db/nation/nis/nisdbdocumentation.jsp.
