# Supplementary material for: Trends and patterns in pulmonary arterial hypertension-associated hospital admissions among methamphetamine users: a decade-long study
Source: Front Cardiovasc Med. 2024 Oct 28;11:1445193. doi: 10.3389/fcvm.2024.1445193 (PMC11550945; doi:10.3389/fcvm.2024.1445193)
Supplement: Supplementary file 1 [file Datasheet1.docx]

**Supplemental Information:**

**Table 3:**

| **Inclusionary ICD-9** | **Inclusionary Diagnosis** | **Inclusionary ICD-10** | **Inclusionary Diagnosis** |
| --- | --- | --- | --- |
| 416.0 | Primary PH | I27.0 | Primary PH |
| 416.8 | Other secondary PH | I27.8 | Other specified pulmonary heart disease. No description. |
| 416.1 | Kyphoscoliotic heart disease | I27.1  I27.2 | Kyphoscoliotic heart disease  Other secondary PH |
| 747.3 | Congenital systemic to pulmonary shunt | Q25.72 | Congenital AV pulmonary shunt |
| 710.9 | Unspecified diffuse connective tissue disease | M30-M36 | Systemic connective tissue disorders |
| 416.9 | Chronic pulmonary heart disease, unspecified. Chronic cardiopulmonary disease. Cor pulmonale (chronic) NOS. | I27.9 | Pulmonary heart disease, unspecified. Chronic cardiopulmonary disease. Cor pulmonale (chronic) NOS. |
| 304.4 | Amphetamine Dep NOS | F15.20 | Other stimulant dependence uncomplicated |
| 305.7 | Amphetamine abuse NOS | F15.10 | Other stimulant dependence uncomplicated |

**Table 4:**

| **Exclusionary ICD-9** | **Exclusionary Diagnosis** | **Exclusionary ICD-10** | **Exclusionary Diagnosis** |
| --- | --- | --- | --- |
| 428.1 | Left heart failure | I50.1 | Left ventricular failure, unspecified |
| 428.2 | Systolic HF, NOS | I50.20 | Unspecified systolic (congestive) heart failure |
| 428.3 | Diastolic HF, NOS | I50.30 | Unspecified diastolic (congestive) heart failure |
| 424 | Mitral valve disorders | I34 | Nonrheumatic mitral valve disorders |
| 424.1 | Aortic valve disorders | I35 | Nonrheumatic aortic valve disorders |
| 394.9 | Other and unspecified mitral valve diseases | I34.8, I34.9 | Other and unspecified nonrheumatic mitral valve disease |
| 396 | Mitral valve stenosis and aortic valve stenosis | I08.0 | Rheumatic disorders of both mitral and aortic valves |
| 396.1 | Mitral valve stenosis and aortic valve insufficiency | I08.0 | Rheumatic disorders of both mitral and aortic valves |
| 396.2 | Mitral valve insufficiency and aortic valve stenosis | I08.0 | Rheumatic disorders of both mitral and aortic valves |
| 396.3 | Mitral valve insufficiency and aortic valve insufficiency | I08.0 | Rheumatic disorders of both mitral and aortic valves |
| 394 | Mitral stenosis | 105.0, I34.2 | Rheumatic mitral stenosis , nonrheumatic mitral stenosis |
| 395 | Rheumatic aortic stenosis | I06.0 | Rheumatic aortic stenosis |
| 396.8 | Multiple involvement of mitral and aortic valves | 108.8 | Other rheumatic multiple valve diseases |
| 496 | Chronic airway obstruction, NOS | J44.9 | Chronic obstructive pulmonary disease, unspecified |
| 491.21 | Obstructive chronic bronchitis with acute exacerbation | J44.1 | Chronic obstructive pulmonary disease with (acute) exacerbation |
| 495.8 | Other specified allergic alveolitis and pneumonitis | J67.8 | Hypersensitivity pneumonitis due to other organic dusts |
| 495.2 | Bird fancier’s lung | J67.2 | Bird fancier’s lung |
| 508.1 | Chronic and other pulmonary manifestations due to radiation | J70.1 | Chronic and other pulmonary manifestations due to radiation |
| 516.3 | Idiopathic interstitial pneumonia, NOS | J84.111 | Idiopathic interstitial pneumonia, not otherwise specified |
| 503 | Pneumoconiosis due to other inorganic dusts | J63.6 | Pneumoconiosis due to other specified inorganic dusts |
| 515 | Post inflammatory pulmonary fibrosis | J84.10 | Pulmonary fibrosis, unspecified |
| 502 | Pneumoconiosis due to silica or silicates | J62.8 | Pneumoconiosis due to other dust containing silica |
| 748.4 | Congenital cystic lung | Q33.0 | Congenital cystic lung |
| 492.8 | Other emphysema | J43.9 | Emphysema, unspecified |
| 714.81 | Rheumatoid lung | M05.10 | Rheumatoid lung disease with rheumatoid arthritis of unspecified site |
| 327.2 | Organic sleep apnea NOS | G47.3 | Sleep apnea |
| 780.5 | Unspecified sleep disturbance | G47.9 | Sleep disorder, unspecified |
| V12.51 | Personal history of venous thrombosis and embolism | Z86.718 | Personal history of venous thrombosis and embolism |
| 780.57 | Unspecified sleep apnea | G47.30 | Sleep apnea, unspecified |
| 673.2 | Thromboembolism in pregnancy | 088.21 | Thromboembolism in pregnancy |
| 282 | Hereditary spherocytosis | D58.0 | Hereditary spherocytosis |
| 460-5190 | History of chronic respiratory disease | V17.6 | Chronic respiratory condition |
| 415.1 | Pulmonary embolism | I26.9 | Pulmonary embolism |
